# Prognostic value of pulmonary transit time by cardiac magnetic resonance imaging in ST-elevation myocardial infarction

**DOI:** 10.1007/s00330-022-09050-5

**Published:** 2022-08-18

**Authors:** Mathias Pamminger, Martin Reindl, Christof Kranewitter, Felix Troger, Christina Tiller, Magdalena Holzknecht, Ivan Lechner, Paulina Poskaite, Gert Klug, Christian Kremser, Sebastian J. Reinstadler, Bernhard Metzler, Agnes Mayr

**Affiliations:** 1 University Hospital for Radiology, Medical University of Innsbruck, Anichstraße 35, 6020 Innsbruck, Austria; 2 University Hospital for Internal Medicine III (Cardiology and Angiology), Medical University of Innsbruck, Anichstraße 35, 6020 Innsbruck, Austria

**Keywords:** Pulmonary transit time, Prognosis, Cardiac magnetic resonance imaging, ST-segment-elevation myocardial infarction

## Abstract

**Objectives:**

To investigate the prognostic value of pulmonary transit time (pTT) determined by cardiac magnetic resonance (CMR) after acute ST-segment-elevation myocardial infarction (STEMI).

**Methods:**

Comprehensive CMR examinations were performed in 207 patients 3 days and 4 months after reperfused STEMI. Functional parameters and infarct characteristics were assessed. PTT was defined as the interval between peaks of gadolinium contrast time-intensity curves in the right and left ventricles in first-pass perfusion imaging. Cox regression models were calculated to assess the association between pTT and the occurrence of major adverse cardiac events (MACE), defined as a composite of death, re-infarction, and congestive heart failure.

**Results:**

PTT was 8.6 s at baseline and 7.8 s at the 4-month CMR. In Cox regression, baseline pTT (hazard ratio [HR]: 1.58; 95% CI: 1.12 to 2.22; *p* = 0.009) remained significantly associated with MACE occurrence after adjustment for left ventricular ejection fraction (LVEF) and cardiac index. The association of pTT and MACE remained significant also after adjusting for infarct size and microvascular obstruction size. In Kaplan-Meier analysis, pTT ≥ 9.6 s was associated with MACE (*p* < 0.001). Addition of pTT to LVEF resulted in a categorical net reclassification improvement of 0.73 (95% CI: 0.27 to 1.20; *p* = 0.002) and integrated discrimination improvement of 0.07 (95% CI: 0.02 to 0.13; *p* = 0.007).

**Conclusions:**

After reperfused STEMI, CMR-derived pTT was associated with hard clinical events with prognostic information independent of and incremental to infarct size and LV systolic function.

**Key Points:**

*• Pulmonary transit time is the duration it takes the heart to pump blood from the right chambers across lung vessels to the left chambers.*

*• This prospective single-centre study showed inferior outcome in patients with prolonged pulmonary transit time after myocardial infarction.*

*• Pulmonary transit time assessed by magnetic resonance imaging added incremental information to established prognostic markers.*

## Introduction

The evaluation of surrogate markers of cardiopulmonary status may provide risk stratification and prognostication in patients with acute myocardial infarction [1, 2]. While mere physical examination is limited by low sensitivity and poor prognostic value, cardiac catheterisation as the gold standard for haemodynamic congestion evaluation is accompanied by limitations due to its invasive nature [3]. The concept of pulmonary blood flow assessment via imaging modalities to evaluate cardiopulmonary haemodynamic congestion has been described before in both pulmonary and cardiac conditions [4, 5]. Pulmonary transit time (pTT) is defined as the time needed to pump a bolus of blood or, in diagnostics, a bolus of contrast medium from the right ventricle (RV) through the pulmonary vasculature to the left ventricle (LV). PTT is known to be influenced by RV systolic function, pulmonary vasculature resistance and LV diastolic dysfunction [4–6]. It has therefore been described as a quantitative marker for integrative cardiopulmonary status. Various conditions such as chronic heart failure, congenital heart disease and surgical cardiac procedures have been linked to impaired pTT [7–10]. Magnetic resonance imaging has been validated for pulmonary blood volume evaluation based on contrast bolus transit in a phantom [11]. Recently, a large study in patients with known or suspected coronary artery disease who were referred for perfusion cardiac magnetic resonance (CMR) imaging found an association between pTT at rest and nonfatal major adverse cardiovascular events (MACE) independent of conventional risk parameters [12]. However, CMR data on pTT early after ST-elevation myocardial infarction (STEMI) as well as in the follow-up are lacking so far. Moreover, the value of pTT for prediction of MACE following STEMI remains unclear.

We therefore aimed to investigate (1) the dynamics of pTT with progressive infarct healing within the first 4 months after STEMI and (2) the prognostic value of pTT in terms of predicting hard clinical events after STEMI.

## Methods

### Patient population and endpoint definitions

In this prospective observational study at the Heart Center of the University Clinic of Innsbruck, consecutive STEMI patients who met the following inclusion criteria were enrolled: first-time STEMI according to the ESC/ACC/AHA committee criteria [13], revascularisation by primary percutaneous coronary intervention (pPCI) within 24 h after onset of ischaemic symptoms and Killip class < 3 at the time of CMR. The following exclusion criteria were applied: estimated glomerular filtration rate ≤ 30 mL/min per 1.73 m^2^, age < 18 years, any history of coronary artery disease or previous myocardial infarction and general contraindications to perform magnetic resonance imaging.

The primary endpoint of the study was the occurrence of MACE, consisting of all-cause death, myocardial re-infarction and re-hospitalisation for new congestive heart failure. Re-infarction was defined in accordance with the redefined ESC/ACC committee criteria [14]. New congestive heart failure was defined as a first episode of cardiac decompensation after discharge requiring intravenous diuretic therapy [15]. Coronary perfusion before and after pPCI was assessed according to thrombolysis in myocardial infarction (TIMI) grade flow [16]. Clinical endpoints were enquired via telephone interviews by trained personal blinded to baseline data at 6 months, 12 months and 24 months using a standardised questionnaire. All declared endpoints were carefully checked afterwards by reviewing medical records.

All patients gave written informed consent before inclusion. This study was designed and conducted in compliance with the Declaration of Helsinki and approved by the local research ethics committee of the Medical University of Innsbruck.

### Cardiac magnetic resonance imaging

All examinations were performed on a 1.5-T clinical MR imaging unit (MAGNETOM Avanto and Avanto^fit^; Siemens Healthineers) within the first week and 4 months after reperfused STEMI, respectively. Patients were positioned head first in supine position on a multi-channel spine phased-array radiofrequency coil with 24 elements integrated into the patient table. An 18-channel body coil was additionally placed in craniocaudal alignment on the chest of the study subjects.

Our imaging protocol including imaging parameters has been published in detail before [17]. In short, long- and short-axis cine imaging stacks were acquired by retrospective ECG-triggered steady-state free-precession imaging in breath-hold technique. Late gadolinium enhancement imaging for the assessment of infarct size as well as the presence and extent of microvascular obstruction was performed 15 min after intravenous injection of a 0.2-mmol/kg contrast agent bolus (Gadovist; Bayer) by an ECG-triggered phase-sensitive inversion recovery sequence.

Furthermore, for pTT assessment, a first-pass perfusion sequence was added to the protocol, using a cardiac-gated single-shot 2D saturation recovery gradient echo sequence (true fast imaging with steady-state free precession); typical imaging parameters were as follows: repetition time/echo time: 2.2 ms/0.97 ms, saturation recovery time: 90 ms, flip angle: 50°, acquisition matrix: 128 × 82, field of view: 400 × 300 mm, bandwidth: 1370 Hz/pixel, slice thickness: 8 mm, parallel imaging mode: GRAPPA, and acceleration factor: 2. Image acquisition was started simultaneously with contrast media injection; a total of 60 images in one 4-chamber long-axis view as well as two short-axis views were acquired.

### Image post-processing and interpretation

LV and RV volumes and LV mass were assessed on short-axis cine stacks (10–12 slices) using standard software (ARGUS; Siemens Healthineers). Papillary muscles were included to the blood pool [18]. Cardiac output was calculated as the product of LV stroke volume and heart rate. Infarct size and the extent of microvascular obstruction were evaluated via the +5-SD technique where the mean signal intensity of the remote myocardium opposing the infarcted segment was used as reference, as recently described [17, 19].

**Fig. 1:**
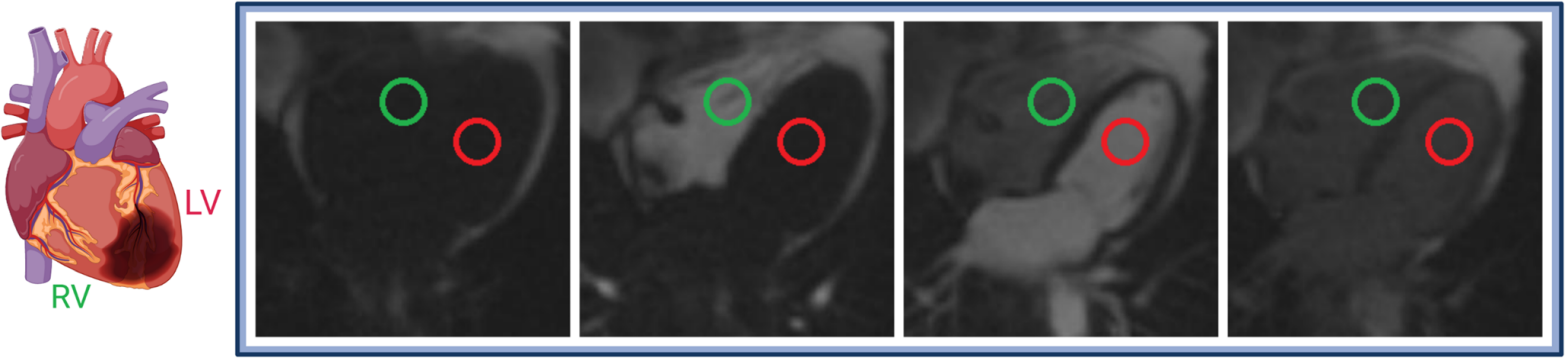
Pulmonary transit time measurement. The figure shows region of interest (ROI) placement within the basis of the right (green circle) and left (red circle) ventricles. The ROI was propagated to all images of the stack and an indicator-dilution curve war created by plotting the average signal intensity within the ROI as a function of time. Subsequent gamma variate fitting of these curves was performed (illustration created using biorender.com)

PTT time-intensity curves were created on syngo.via imaging software (VB40B, Siemens Healthineers), as used in clinical routine. Circular regions of interest (ROI) were placed within the basal RV and LV blood pool on the first-pass perfusion 4-chamber long-axis view. Care was taken to avoid partial volume effects at the papillary muscles or myocardial wall (Fig. 1). ROIs were then propagated to all images of the stack [8]. Average signal intensity within the ROI was plotted as a function of time, resulting in an indicator-dilution curve. Gamma variate fit was performed using the R Project for Statistical Computing 4.0.5 software (R Foundation for Statistical Computing) to obtain reliable time to peak values [20]. Of note, gamma variate fit only models the initial intensity peak; consecutive regional peaks due to recirculation were not modelled. PTT was defined as the interval between peaks of the RV and LV fit curves, which delivered the most reliable pTT estimates in our cohort [5]. Pulmonary blood volume index (PBVI) was calculated using volumetric cardiac output (mL/min) as:

$$ PBVI=\frac{cardiac\ output}{60}\ast pTT\ast \frac{1}{body\ surface\ area} $$

All CMR images were analysed by experienced observers blinded to clinical information and previous examinations in the case of follow-up examinations. A subset of 20 patients was randomly selected for determination of inter- and intra-observer variability.

### Statistical analysis

SPSS Statistics 27.0 (IBM) and R 4.1.2 (R Foundation for Statistical Computing) were used for statistical analysis. Continuous variables were expressed as median with interquartile range (IQR), categorical variables as absolute number and corresponding percentage. Differences in continuous variables between two groups were evaluated by Mann-Whitney *U* test; differences in proportions were compared by chi-squared test or Fisher’s exact test, as appropriate. Intra-class correlation coefficients (ICC) by a two-way mixed model for single measures were used to evaluate inter- and intra-observer reliability; differences were tested with Wilcoxon rank test. Univariable and multivariable Cox regression analysis was used for evaluation of significant and independent predictors of MACE. Variables with a *p* value < 0.10 in univariable analysis were included in multivariable models. To ensure statistical robustness with respect to our sample size and event rate, two different models were defined a priori. Apart from pTT, model 1 included dynamic parameters (LVEF to embrace systolic function and cardiac index to additionally embrace heart rate [9]) and was termed “functional model”. Model 2 included infarct parameters (infarct size and microvascular obstruction size, each relative to LV myocardial mass (LVMM)) and was therefore termed “tissue model”. Receiver operating characteristic analysis with Youden’s *J* statistic was performed to determine optimal cut-off values [21]. MACE-free survival was assessed by the Kaplan-Meier method; differences between groups were computed by the log-rank test. To evaluate the incremental prognostic benefit of pTT over LVEF for MACE prediction, net reclassification improvement and integrated discrimination improvement were calculated by using the R package “PredictABEL”. The optimal cut-off values for pTT, infarct size and microvascular obstruction size according to Youden’s *J* statistic and LVEF ≤ 40% as established cut-off value for additional medical treatment to reduce the risk of cardiovascular hospitalisation and death were used for calculation [14]. A two-tailed *p* value of < 0.05 was considered statistically significant.

## Results

### Patient characteristics

**Fig. 2:**
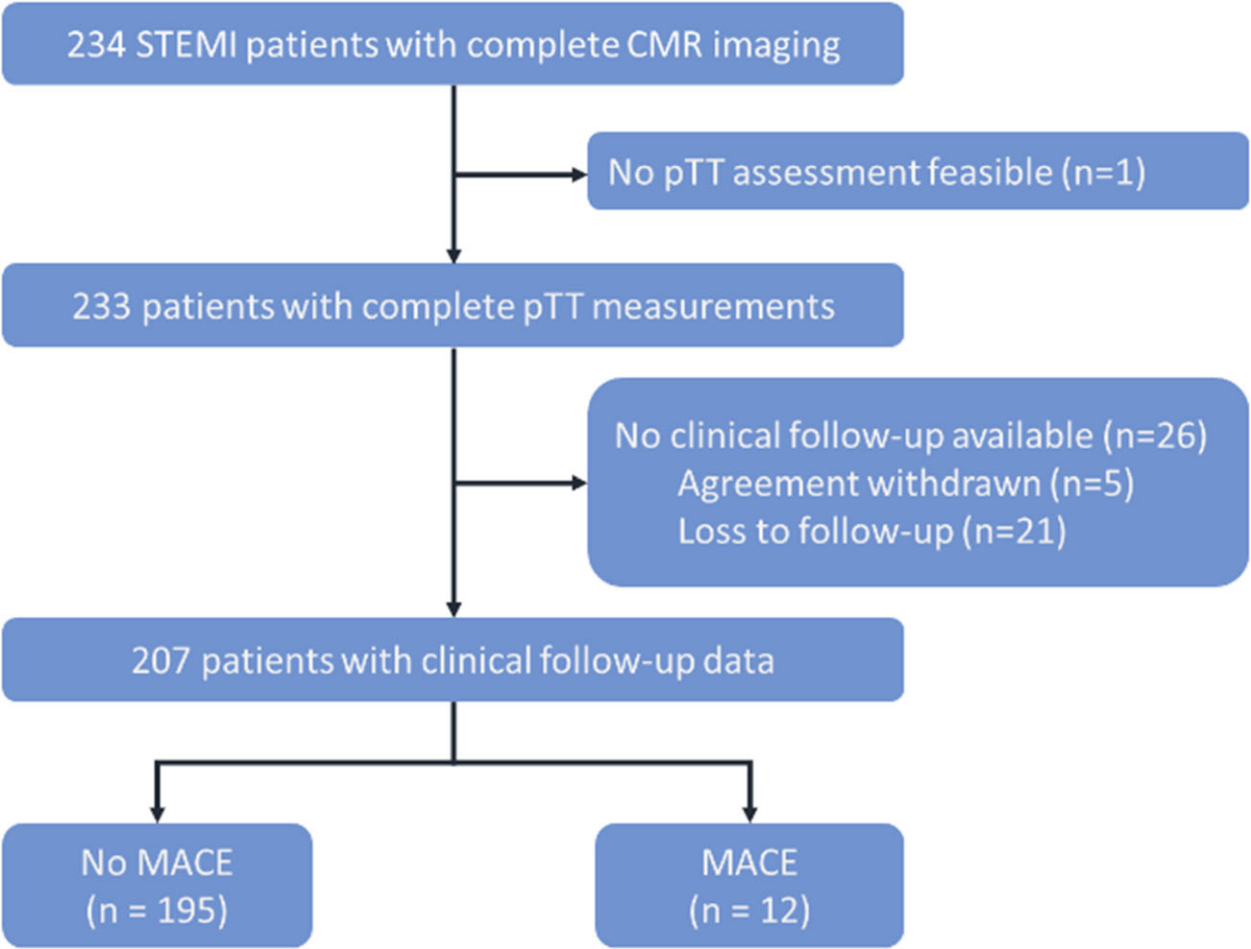
Study flow chart. Abbreviations: CMR = cardiac magnetic resonance; MACE = major adverse cardiac events; pTT = pulmonary transit time; STEMI = ST-segment-elevation myocardial infarction

In total, 207 STEMI patients were included in the final analysis. A detailed study flow chart is depicted by Fig. 2. Median age of the overall cohort was 55 (IQR: 49 to 64) years and 28 patients (14%) were female. Left ventricular thrombus was found in 3 (1.5%) patients. Of 83 patients with right coronary artery infarction, 22 (27%) had right ventricular late gadolinium enhancement involvement. Detailed baseline patient characteristics are provided in Table 1.

**Table 1:** Comparison of patient baseline characteristics

Characteristics	Total population (*n* = 207)	PTT ≥ 9.6 s (*n* = 53)	PTT < 9.6 s (*n* = 154)	*p* value
Age, years	55 (49–64)	60 (50–69)	55 (49–63)	0.074
Female, *n* (%)	28 (14)	5 (9)	23 (15)	0.312
Body mass index, kg/m^2^	26.1 (24.2–28.4)	25.5 (24.0–27.5)	26.2 (24.3–28.8)	0.111
Peak hs-cTnT, ng/L	5330 (2430–8182)	6786 (3169–9223)	4658 (2273–7598)	**0.012**
Cardiovascular risk factors, *n* (%)				
Diabetes mellitus	21 (10)	4 (8)	17 (11)	0.468
Hyperlipidemia	141 (68)	32 (60)	109 (71)	0.161
Current smoker	118 (59)	28 (53)	90 (58)	0.477
Hypertension	123 (59)	97 (63)	26 (49)	0.075
Positive family history	57 (28)	11 (21)	46 (30)	0.200
Total ischaemic time, min	195 (127–347)	210 (120–329)	194 (127–361)	0.801
Culprit lesion, *n* (%)				0.085
RCA	88 (43)	17 (32)	71 (46)	
LAD	83 (40)	28 (53)	55 (36)	
LCX	36 (17)	8 (15)	28 (18)	
Pre-interventional TIMI flow, *n* (%)				0.202
0	133 (64)	38 (72)	95 (62)	
1	31 (15)	4 (8)	27 (18)	
2	39 (19)	11 (21)	28 (18)	
3	4 (2)	0 (0)	4 (3)	
Post-interventional TIMI flow, *n* (%)				0.448
0	6 (3)	3 (6)	3 (2)	
1	3 (1)	1 (2)	2 (1)	
2	19 (9)	6 (11)	13 (8)	
3	179 (86)	43 (81)	136 (88)	

Baseline characteristics of the overall population and in groups, dichotomised by the optimal cut-off pulmonary transit time (pTT) at baseline CMR examination. Values are either *n* (%) or median (IQR). Statistically significant *p* values are printed in bold

*hs-cTnT* high-sensitivity cardiac troponin T, *LAD* left anterior descending artery, *LCX* left circumflex artery, *pTT* pulmonary transit time, *RCA* right coronary artery, *TIMI* thrombolysis in myocardial infarction

### Cardiac MRI parameters

CMR scans were performed at a median of 3 (IQR: 2 to 4) days and 18 (IQR: 17 to 20) weeks after STEMI. In the overall population, pTT was 8.6 seconds (s) (IQR: 7.5 to 9.7 s) at baseline and decreased to 7.8 s (IQR: 7.1 to 8.7 s) within the first 4 months after STEMI (*p* < 0.001).

**Fig. 3:**
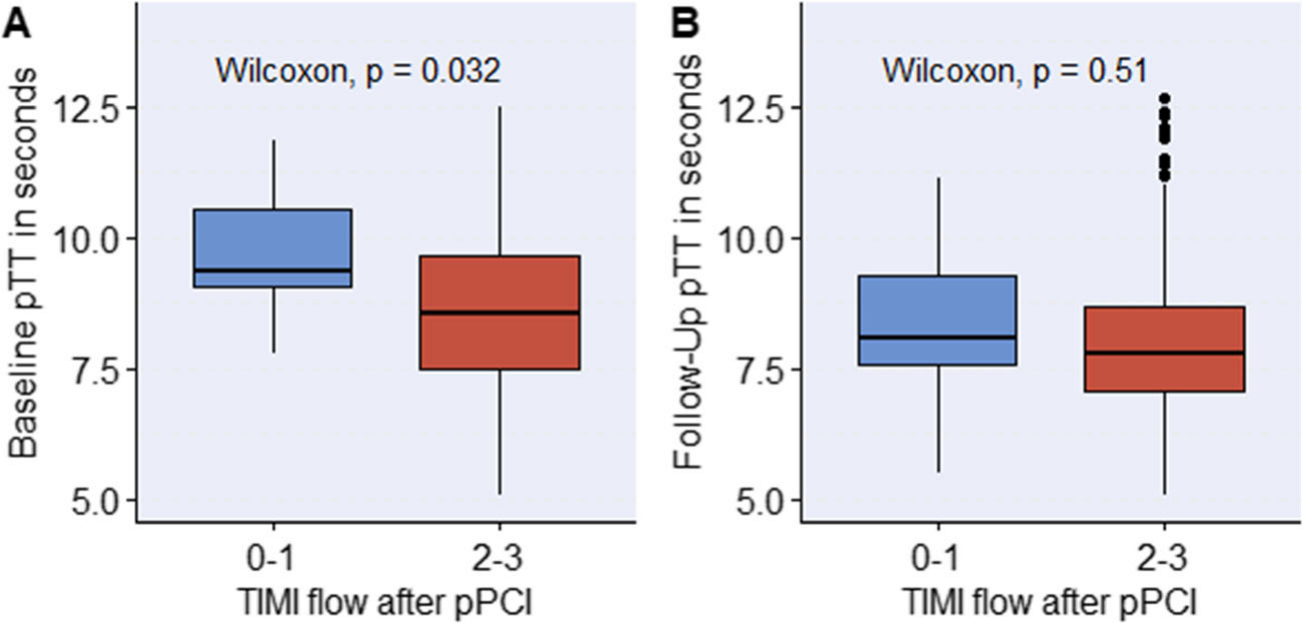
Pulmonary transit time according to interventional outcome. PTT at baseline (**A**) and 4-month follow-up (**B**) according to post-interventional TIMI grade flow. Patients with inferior interventional outcome showed prolonged pTT at baseline; however, differences in pTT diminished within the first 4 months. Abbreviations: pTT = pulmonary transit time, pPCI = primary percutaneous coronary intervention, TIMI = thrombolysis in myocardial infarction

Imaging parameters at baseline and follow-up CMR in the overall population and dichotomised by the optimal pTT cut-off value are summarised in Table 2. Patients with inferior (score 0 and 1) interventional results according to TIMI flow (11 patients; 6%) showed prolonged pTT at baseline (9.3 s; IQR: 9.1 to 10.5 s vs 8.5 s; IQR: 7.5 to 9.7 s; *p* = 0.032), but not at follow-up (Fig. 3).

**Table 2:** Comparison of patient imaging parameters

Characteristics	Total population (*n* = 207)	PTT ≥ 9.6 s (*n* = 53)	PTT < 9.6 s (*n* = 154)	*p* value
CMR parameters at baseline				
LV ejection fraction, %	54 (47–60)	50 (42–54)	55 (49–61)	**< 0.001**
LV end-diastolic volume, mL	152 (126–168)	164 (139–180)	147 (121–165)	**0.003**
LV end-systolic volume, mL	68 (51–83)	80 (61–108)	63 (48–79)	**< 0.001**
LV stroke volume, mL	78 (66–92)	78 (63–88)	77 (66–92)	0.427
Cardiac index, L/min per m^2^	2.9 (2.4–3.2)	2.4 (2.2–2.9)	3.0 (2.6–3.3)	**< 0.001**
RV ejection fraction, %	57 (51–62)	56 (49–62)	57 (52–62)	0.314
RV end-diastolic volume, mL	143 (120–165)	144 (119–166)	143 (122–164)	0.939
RV end-systolic volume, mL	61 (48–81)	64 (46–84)	60 (48–76)	0.550
RV stroke volume, mL	81 (64–93)	77 (60–93)	81 (65–95)	0.337
PTT, s	8.6 (7.5–9.7)	10.7 (10.2–11.5)	8.0 (7.1–8.8)	**< 0.001**
PBVI, mL per m^2^	401 (345–464)	450 (399–496)	383 (334–443)	**< 0.001**
Infarct size, % of LVMM	14 (8–22)	16 (8–25)	13 (7–21)	0.181
Microvascular obstruction, *n* (%)	117 (57)	37 (70)	82 (53)	**0.045**
Microvascular obstruction, % of LVMM	0.4 (0.0–2.1)	1.2 (0.0–3.9)	0.3 (0.0–1.9)	**0.022**
CMR parameters at follow-up				
LV ejection fraction, %	58 (50–64)	53 (44–61)	59 (51–65)	**0.001**
LV end-diastolic volume, mL	150 (125–173)	160 (142–182)	146 (121–169)	**0.001**
LV end-systolic volume, mL	60 (49–84)	75 (58–103)	57 (46–77)	**< 0.001**
LV stroke volume, mL	84 (71–96)	85 (69–96)	84 (72–97)	0.966
Cardiac index, L/min per m^2^	2.8 (2.4–3.1)	2.5 (2.2–2.9)	2.8 (2.5–3.1)	**0.003**
RV ejection fraction, %	54 (50–59)	53 (49–60)	54 (50–58)	0.964
RV end-diastolic volume, mL	140 (119–165)	146 (122–170)	137 (119–163)	0.320
RV end-systolic volume, mL	63 (53–79)	66 (55–80)	62 (53–79)	0.391
RV stroke volume, mL	75 (63–87)	79 (63–86)	72 (63–89)	0.594
PTT, s	7.8 (7.1–8.7)	8.6 (7.6–10.5)	7.6 (6.9–8.5)	**< 0.001**
PBVI, mL per m^2^	359 (310–407)	368 (314–436)	355 (310–402)	0.490
Infarct size, % of LVMM	11 (6–17)	14 (7–21)	10 (6–16)	**0.043**

Imaging parameters of the overall population at baseline and 4-month follow-up as well as in groups, dichotomised by the optimal cut-off pulmonary transit time (pTT) at baseline CMR examination. Values are either *n* (%) or median (IQR). Statistically significant *p* values are printed in bold

*CMR* cardiac magnetic resonance, *LV* left ventricular, *LVMM* left ventricular myocardial mass, *PBVI* pulmonary blood volume index, *pTT* pulmonary transit time, *RV* right ventricular

### Prognostic value of pTT

During a median follow-up time of 25 (IQR: 24 to 25) months, 12 patients (6%) suffered from MACE (3 deaths, 4 re-infarctions and 5 heart failure events). Patients with MACE had significantly longer baseline pTT (10.1 s; IQR: 8.3 to 11.3 s vs 8.5 s; IQR: 7.5 to 9.5 s; *p* = 0.009) and lower LVEF at baseline (46%; IQR: 32 to 57% vs 54%; IQR: 47 to 60%; *p* = 0.031) and at 4-month follow-up (46%; IQR: 39 to 61% vs 59%; IQR: 50 to 64%; *p* = 0.017) as well as a larger extent of microvascular obstruction (3.1% of LVMM; IQR: 0.6 to 6.3% vs 0.4% of LVMM; IQR: 0.0 to 2.0%; *p* = 0.007). PBVI was comparable in patients with and without MACE (438 mL; IQR: 375 to 489 mL vs 400 mL; IQR: 343 to 457 mL; *p* = 0.130).

In a multivariable model (“functional model”) including pTT, LVEF and cardiac index only pTT (hazard ratio (HR): 1.58; 95% confidence interval (CI): 1.12 to 2.22; *p* = 0.009) significantly predicted MACE. As demonstrated by a multivariable “tissue model”, pTT (HR: 1.46; 95% CI: 1.02 to 2.10; *p* = 0.038) and microvascular obstruction size (HR: 1.20; 95% CI: 1.08 to 1.32; *p* = 0.001) remained significant predictors of MACE after adjustment for infarct size (Table 3). PBVI did not significantly predict MACE (HR: 1.00; 95% CI 1.00 to 1.01; *p* = 0.091).

**Table 3:** Cox logistic regression models

Variable at baseline	Univariable analysis		Multivariable analysis	
	Hazard ratio (95% CI)	*p* value	Hazard ratio (95% CI)	*p* value
Functional model				
PTT, s	1.58 (1.12–2.22)	**0.009**	1.58 (1.12–2.22)	**0.009**
LV ejection fraction, %	0.94 (0.90–0.98)	**0.008**		
Cardiac index, L/min per 1.73 m^2^		0.443		
Tissue model				
PTT, s	1.58 (1.12–2.22)	**0.009**	1.46 (1.02–2.10)	**0.038**
Infarct size, % of LVMM	1.05 (1.01–1.09)	**0.002**		
Microvascular obstruction, % of LVMM	1.20 (1.09–1.32)	**< 0.001**	1.20 (1.08–1.32)	**0.001**

Cox logistic regression models for prediction of major adverse cardiac events (MACE). To ensure statistical robustness with respect to our event rate (12 MACE), two models were created. Statistically significant *p* values are printed in bold

*CI* confidence interval, *LV* left ventricular, *LVMM* left ventricular myocardial mass, *pTT* pulmonary transit time

**Fig. 4:**
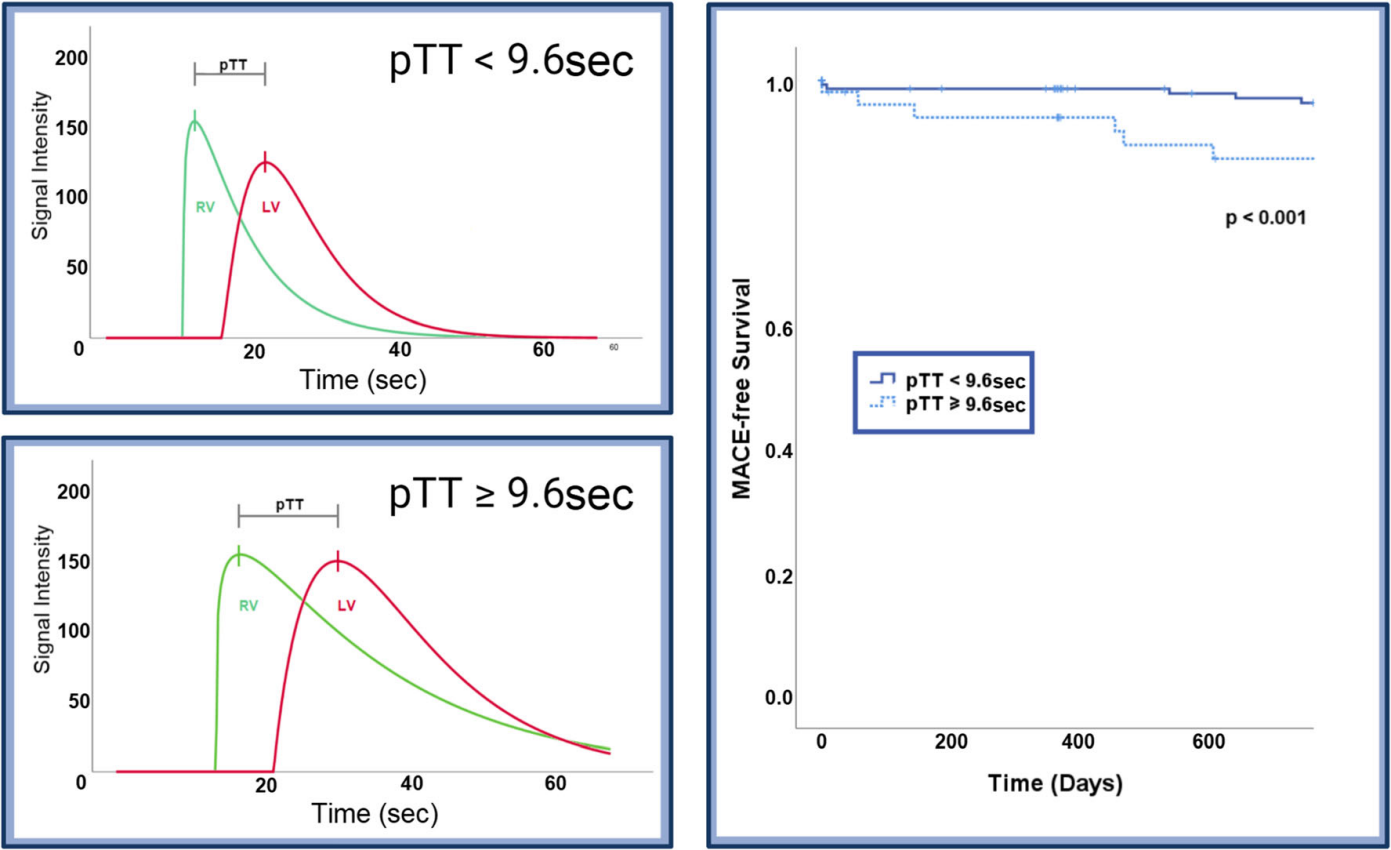
Pulmonary transit time and survival. The two left panels show average fit curves for patients with pTT below the optimal cut-off value (9.6 s) for MACE prediction and above the optimal cut-off. Event-free survival within 24 months for groups dichotomised by the optimal cut-off (9.6 s) is shown in the right panel with a higher event rate in patients with prolonged pTT. Abbreviations: LV = left ventricle; MACE = major adverse cardiac events; pTT = pulmonary transit time; RV = right ventricle

The best pTT cut-off value for MACE prediction according to Youden’s *J* statistic was 9.6 s. According to the Kaplan-Meier analysis, patients with baseline pTT ≥ 9.6 s showed significantly lower MACE-free survival (*p* < 0.001) than patients with pTT < 9.6 s (Fig. 4). Addition of prolonged pTT (≥ 9.6 s) to reduced LVEF (≤ 40%) resulted in a categorical net reclassification improvement (0.03, 0.06, 0.12) of 0.73 (95% CI: 0.27 to 1.20; *p* = 0.002), continuous net reclassification improvement of 1.03 (95% CI: 0.52 to 1.53; *p* < 0.001) and integrated discrimination improvement of 0.07 (95% CI: 0.02 to 0.13; *p* = 0.007). The addition of prolonged pTT to a risk model based on infarct size (optimal cut-off 21% of LVMM) resulted in a categorical net reclassification improvement (0.03, 0.06, 0.12) of 1.03 (95% CI: 0.52 to 1.53; *p* < 0.001), continuous net reclassification improvement of 1.03 (95% CI: 0.52 to 1.53; *p* < 0.001) and integrated discrimination improvement of 0.08 (95% CI: 0.04 to 0.11; *p* < 0.001).

### Inter-observer and intra-observer variability of pTT measurements

The inter-observer difference of pTT was 0.1 (IQR: − 0.1 to 0.2) s with an ICC of 0.96 (95% CI: 0.89 to 0.98; *p* < 0.001). There was no relevant intra-observer variability (mean difference of 0.0 (IQR: − 0.1 to 0.1) s with an ICC of 0.96 (95% CI: 0.90 to 0.98; *p* < 0.001)).

## Discussion

This study is the first to assess pTT as well as its prognostic value in a large cohort of acute STEMI patients, homogenously treated by pPCI. The main findings can be summarised as follows:

1. PTT, as assessed by contrast-enhanced first-pass perfusion CMR, decreases significantly within the first 4 months after reperfused STEMI.
2. Prolonged baseline pTT predicts hard clinical events independent of infarct size and LV systolic function.
3. Addition of pTT to LVEF or infarct size led to improved risk stratification regarding MACE post-STEMI.

To summarise, pTT derived by first-pass perfusion CMR independently enables risk assessment after STEMI and expands the prognostic significance of LVEF and infarct size.

Previous CMR studies reported pTT being prolonged in patients with heart failure and congenital heart disease and after the Ross procedure as compared to healthy controls [7, 9, 10]. Ricci et al reported prolonged pTT in patients compared to healthy volunteers in an inhomogeneous group of 112 heart failure patients of which roughly 50% suffered from ischaemic cardiomyopathy, and associated pulmonary blood volume index with adverse outcome [5]. These previous studies measured pTT from the RV to the left atrium and the LV, respectively, from the right atrium to the ascending aorta and from the pulmonary artery to the ascending aorta. Transit time was found to be influenced by RV systolic function, pulmonary vasculature and LV diastolic function [4, 5, 7]. We deliberately defined pTT as RV to LV transit time, which, beyond considering the influence of RV function and pulmonary circulation, integrates overall LV function to a certain extent as well. Furthermore, assessment on a standard 4-chamber long-axis view is feasible. In a preliminary evaluation, we found signal intensities in the right atrium to markedly vary, in some cases prohibiting sufficient fitting of gamma variate functions. Therefore, we decided to position the right sided ROI within the RV, resulting in usable measurements in all but one patient (0.5%).

Although this study is the first to assess pTT after reperfused STEMI in humans, Lin et al evaluated pTT in a mouse model, using optoacoustic imaging for transit time measurement and CMR imaging for assessment of infarct size and LVEF [22]. They reported prolonged transit time dependent on infarct size in the ischaemia model, compared to non-infarcted mice, and gradual improvement over time, which is in line with our finding of pTT decrease within the first 4 months. Although pTT and infarct size at baseline were not associated in our study, infarct size 4 months after infarction was larger in patients with prolonged baseline pTT. A possible explanation may be overestimation of the actual infarct size in CMR examinations in the acute phase, a previously described effect due to concomitant tissue oedema, which resolved by the time of follow-up examination [23].

Another animal study compared effects of sustained coronary occlusion to short-term occlusion as a model for infarct reperfusion [24]. Temporal occlusion resulted in less prolonged transit times compared to sustained occlusion. In our study, all patients underwent pPCI, as modelled by temporal occlusion in the aforementioned study. However, interventional success has been assessed according to TIMI flow in our study. Baseline pTT was longer in patients with inferior post-interventional TIMI score. Of note, pTT improvement was more pronounced in patients with worse interventional outcome. A possible explanation may be additional medical therapy in the clinical setting to improve patient benefit and possibly support normalisation of pTT.

So far, no CMR study investigated pTT in STEMI patients and its link to clinical outcome. The overall median pTT in our study cohort of acute STEMI patients was 8.6 s and thus marginally lower than reported in previous studies with a median pTT of 9 s in heart failure patients and a mean pTT of 9.6 ± 1.6 s after Ross procedure [5, 10]. However, the optimal prognostic discrimination for adverse outcome in our STEMI cohort was at 9.6 s, which further supports a cut-off value of 9–10 s to reasonably define prolonged transit time in various cardiac conditions. Further studies in larger patient cohorts are desirable, before cut-off values for use in clinical routine can be established.

Previous studies have evaluated the prognostic value of PBVI in heart failure, in patients referred for perfusion CMR or after Ross procedure [5, 10, 12]. Although patients with MACE exhibited slightly larger PBVI in our cohort, differences did not reach statistical significance and PBVI was not an independent predictor of MACE. We evaluated left atrial volume in a subgroup of 135 patients with 7 MACE in the subgroup, to adjust for left atrial size variation, as the left atrium contributes to the total PBVI with our method of PTT measurement [25]. Even after correction for left atrial volumes in the subgroup, PBVI did not significantly predict MACE; however, the number of events in subgroup analysis might be too little to draw conclusions.

Generally, a strong trend away from mere assessment of LVEF for risk stratification towards a broader evaluation of cardiac function and tissue characteristics is evident [17, 26]. Especially functional parameters, as which pTT can also be understood, were in the focus of recent efforts to establish new prognostic parameters [27, 28]. This study revealed pTT as the integrative functional parameter to be associated with adverse patient outcome post-STEMI. Similar findings have been shown in patients with congenital heart disease and known or suspected coronary artery disease, suggesting pTT to be of prognostic value in various cardiac conditions other than acute events [9, 12]. Especially in combination with LVEF as a parameter of LV systolic function, the comprehensive cardiac function assessment by pTT improved risk stratification, potentially guiding post-discharge management of STEMI patients.

Recent approaches on pTT quantification mainly focused on echocardiography and CMR imaging, as they both provide non-invasive time-resolved visualisation of a contrast agent bolus in right and left cardiac chambers while crossing the pulmonary vasculature [8, 29]. Although a systematic difference in pTT between CMR imaging and echocardiography of 1.4 s has been described, both methods were shown to reasonably correlate [8]. However, beyond ventricular function and myocardial strain, which can both be assessed by echocardiography as well, comprehensive CMR protocols provide additional tissue characterisation in the same examination to assess myocardial oedema, infarct size and microvascular injury which can help to further predict patient outcome [30]. As pTT can be assessed by first-pass perfusion CMR imaging, a sequence that is already often included in comprehensive CMR protocols, imaging duration does not lengthen, which makes pTT a feasible and readily available parameter. We acknowledge that gamma variate fitting requires some post-processing; however, corresponding automated tools could be implemented in standard CMR reporting software to provide a usable and fast application [12].

We acknowledge the following limitations of our present study:

In this observational CMR study, only stable patients (Killip class < 3) at the time of CMR and first-time STEMI were included; hence, our findings may not be transferable to haemodynamically less stable patients or to patients with previous myocardial infarction. Notably, the vast majority of STEMI patients present with Killip class < 3 [31].

The lack of a validation cohort and the relatively small number of events in our cohort represent an important limitation of the study. Our cohort comprised patients with relatively high LVEF; a subgroup analysis of patients with impaired LVEF was not feasible due to the small number of adverse events. However, pTT was still predictive of patient outcome, highlighting its value for risk stratification even in preserved LV function.

According to current guidelines, echocardiography is the recommended imaging modality during the hospital stay after STEMI, while CMR is considered as an alternative in inconclusive cases, thus hampering the broad applicability of pTT measurement via CMR [14]. However, growing data on improved prognosis assessment with CMR in addition to known advantages of CMR over echocardiography such as better detection of left ventricular thrombus might strengthen the role of CMR after STEMI in future guidelines [32]. As pTT provided incremental prognostic information over LVEF, CMR might be indicated in patients with reduced LVEF in echocardiography; however, further studies to assess possible patient benefit from such an approach are needed.

To conclude, pTT assessed by first-pass perfusion CMR imaging emerged as an independent predictor of MACE with incremental prognostic value to established prognostic parameters and could be used as an alternative for risk stratification after STEMI.

## Abbreviations

CI: Confidence interval
CMR: Cardiac magnetic resonance
HR: Hazard ratio
ICC: Intra-class correlation coefficient
IQR: Interquartile range
LV: Left ventricle/left ventricular
LVEF: Left ventricular ejection fraction
LVMM: Left ventricular myocardial mass
MACE: Major adverse cardiac events
PBVI: Pulmonary blood volume index
pPCI: Primary percutaneous coronary intervention
pTT: Pulmonary transit time
RV: Right ventricle/right ventricular
s: Seconds
STEMI: ST-segment-elevation myocardial infarction

## Declarations

### Guarantor

The scientific guarantor of this publication is Agnes Mayr, MD.

### Statistics and biometry

No complex statistical methods were necessary for this paper.

### Methodology

• prospective

• diagnostic or prognostic study

• performed at one institution
